# Supplementary material for: Micelle Formation in Aqueous Solutions of the Cholesterol-Based Detergent Chobimalt Studied by Small-Angle Scattering
Source: Molecules. 2023 Feb 14;28(4):1811. doi: 10.3390/molecules28041811 (PMC9960369; doi:10.3390/molecules28041811)
Supplement: Supplementary file 1 [file molecules-28-01811-s001.zip › molecules-2167814-supplementary.pdf]

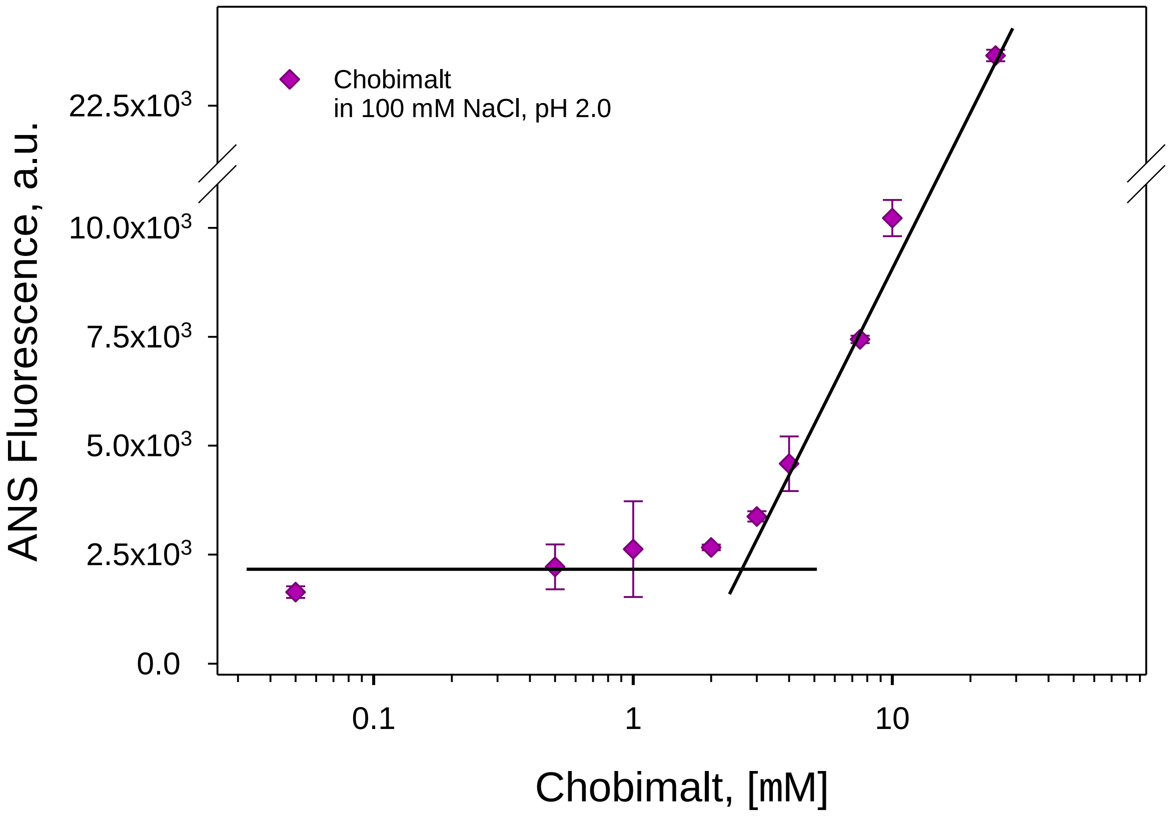

Figure S1. Determination of the critical micellar concentration of Chobimalt using ANS fluorescence assay dissolved in 100 mM NaCl, pH 2.0. The symbols represent the experimental data and the black solid lines are fitting linear curves. The abrupt change in the slope of the surface tension values determines the surfactant critical micelle concentrations (CMC) (CMC is determined as the intersection point of the two lines). The measurements were performed at 20 °C.
